# *In vivo* efficient maternal haploid production in melon via knockout of *CmDMP* genes

**DOI:** 10.1093/plphys/kiag552

**Published:** 2026-08-05

**Authors:** Lu Zhang, Yaomei Zhang, Jiaxi Yang, Yansheng Bi, Huijun Zhang, Qunfeng Lou, Chuntao Qian, Jinfeng Chen

**Affiliations:** State Key Laboratory of Crop Genetics & Germplasm Enhancement and Utilization, College of Horticulture, Nanjing Agricultural University, Nanjing 210095, China; State Key Laboratory of Crop Genetics & Germplasm Enhancement and Utilization, College of Horticulture, Nanjing Agricultural University, Nanjing 210095, China; State Key Laboratory of Crop Genetics & Germplasm Enhancement and Utilization, College of Horticulture, Nanjing Agricultural University, Nanjing 210095, China; State Key Laboratory of Crop Genetics & Germplasm Enhancement and Utilization, College of Horticulture, Nanjing Agricultural University, Nanjing 210095, China; College of Life Sciences, Huaibei Normal University, Huaibei 235000, China; State Key Laboratory of Crop Genetics & Germplasm Enhancement and Utilization, College of Horticulture, Nanjing Agricultural University, Nanjing 210095, China; State Key Laboratory of Crop Genetics & Germplasm Enhancement and Utilization, College of Horticulture, Nanjing Agricultural University, Nanjing 210095, China; State Key Laboratory of Crop Genetics & Germplasm Enhancement and Utilization, College of Horticulture, Nanjing Agricultural University, Nanjing 210095, China

Dear Editor,

Melon (*Cucumis melo* L.) is a horticulturally important crop worldwide. Progress in breeding and genetic research has been impeded by high genomic heterozygosity, poor trait pyramiding, and slow pure-line development. Doubled haploid (DH) technology can produce completely homozygous lines in just 2 generations, thus shortening the breeding cycle in many crops. Though haploid plants can be produced in melon by in vitro techniques, no efficient in vivo haploid induction system can be used for practical DH breeding (Hooghvorst et al. 2020). Recent studies have revolutionized traditional haploid induction breeding by finding and using key genes such as *MTL/NLD/ZmPLA1, DMP, PLD3, POD65, KPL, CENH3,* and *BBM* (Gilles et al. 2017; Kelliher et al. 2017; Liu et al. 2017; Zhong et al. 2019; Li et al. 2021; Jiang et al. 2022; Jacquier et al. 2023; Yang et al. 2025; Shi et al. 2025). However, orthologs of *MTL/NLD/ZmPLA1* have not been identified in dicots, and haploid induction systems based on *CENH3, BBM, POD65, PLD3,* or *KPL* have so far succeeded only in a limited number of crop species (Li et al. 2025). In contrast, *ZmDMP-*like genes are conserved in dicots. Mutations in putative *DMP* orthologs in Arabidopsis (*Arabidopsis thaliana*), tomato (*Solanum lycopersicum*), *Brassica napus*, *Medicago truncatula*, watermelon (*Citrullus lanatus*), cucumber (*Cucumis sativus*), cotton (*Gossypium hirsutum*), *Brassica juncea,* and other dicot species can establish a heritable, genotype-independent in vivo haploid induction system (Table S1; Zhong et al. 2020, 2022; Li et al. 2022; Wang et al. 2022; Tian et al. 2023; Yin et al. 2024; Long et al. 2024; Chu et al. 2025). Nevertheless, whether this system can be effectively applied to melon remains unknown.

To study the potential of *DMP* in melon haploid induction, we identified 8 candidate genes (Table S2) and constructed a phylogenetic analysis (Figure S1). Results showed CmDMP8 (MELO3C010247) and CmDMP9 (MELO3C023229) clustered with functionally characterized DMPs from other species, with high amino acid sequence identity with ZmDMP (65.22% and 66.69%, respectively), and were most closely related to CmDMP8/9 orthologs in cucumber, watermelon, and Arabidopsis, suggesting a conserved role for CmDMP8/9 in haploid induction. Subcellular localization analysis in *Nicotiana benthamiana* epidermal cells showed that both proteins were predominantly localized to the plasma membrane, consistent with other known DMP homologs in crops (Figure S2). We next studied *CmDMP8* and *CmDMP9* expression in melon using RT-qPCR. Both genes were highly expressed in mature anthers and pollen, and *CmDMP9* expression was significantly higher than that of *CmDMP8,* suggesting that this gene could play an important role in late gametophyte development in melon (Figure S3).

To investigate the role of *CmDMP8* and *CmDMP9* in melon haploid induction, we generated single and double knockout mutants using a CRISPR-Cas9 system based on the pKSE402 binary vector (Fig. 1a). This vector contains a distinct eGFP marker expression cassette driven by the CaMV 35S promoter, and 2 pairs of gene-specific gRNAs targeting *CmDMP8* and *CmDMP9*. Constructed plasmids were transformed into the local melon cultivar ‘M15' via *Agrobacterium*-mediated transformation. In the T_1_ generation, we successfully identified CRISPR/Cas9-edited mutants carrying frameshift-inducing deletions or insertions at the *CmDMP8* and/or *CmDMP9* loci (Fig. 1b; Table S3). The *cmdmp8cmdmp9* double mutants exhibited no obvious vegetative phenotypic differences compared with the wild type (WT) under greenhouse or field conditions (Figure S4). Notably, they displayed severely compromised fertility, with low pollen viability, reduced seed set, and increased seed abortion (Fig. 1c–g), consistent with the previously implicated roles of *CmDMP8* and *CmDMP9* in fertilization (Jacquier et al. 2020). Haploid melon plants, displaying typical dwarfism, narrow leaves, small ovaries, and pollen sterility, were identified among the self-pollinated progeny of the *cmdmp8cmdmp9* double mutants (Fig. 1h and i). The average haploid induction rate (HIR) in their T_1_ progeny was 0.59% (14/2367), compared to 0.33% (7/2144) in *cmdmp9* single mutants. In contrast, no haploids were detected in the progeny of *cmdmp8* single mutants or WT plants (Table S4).

**Figure 1:**
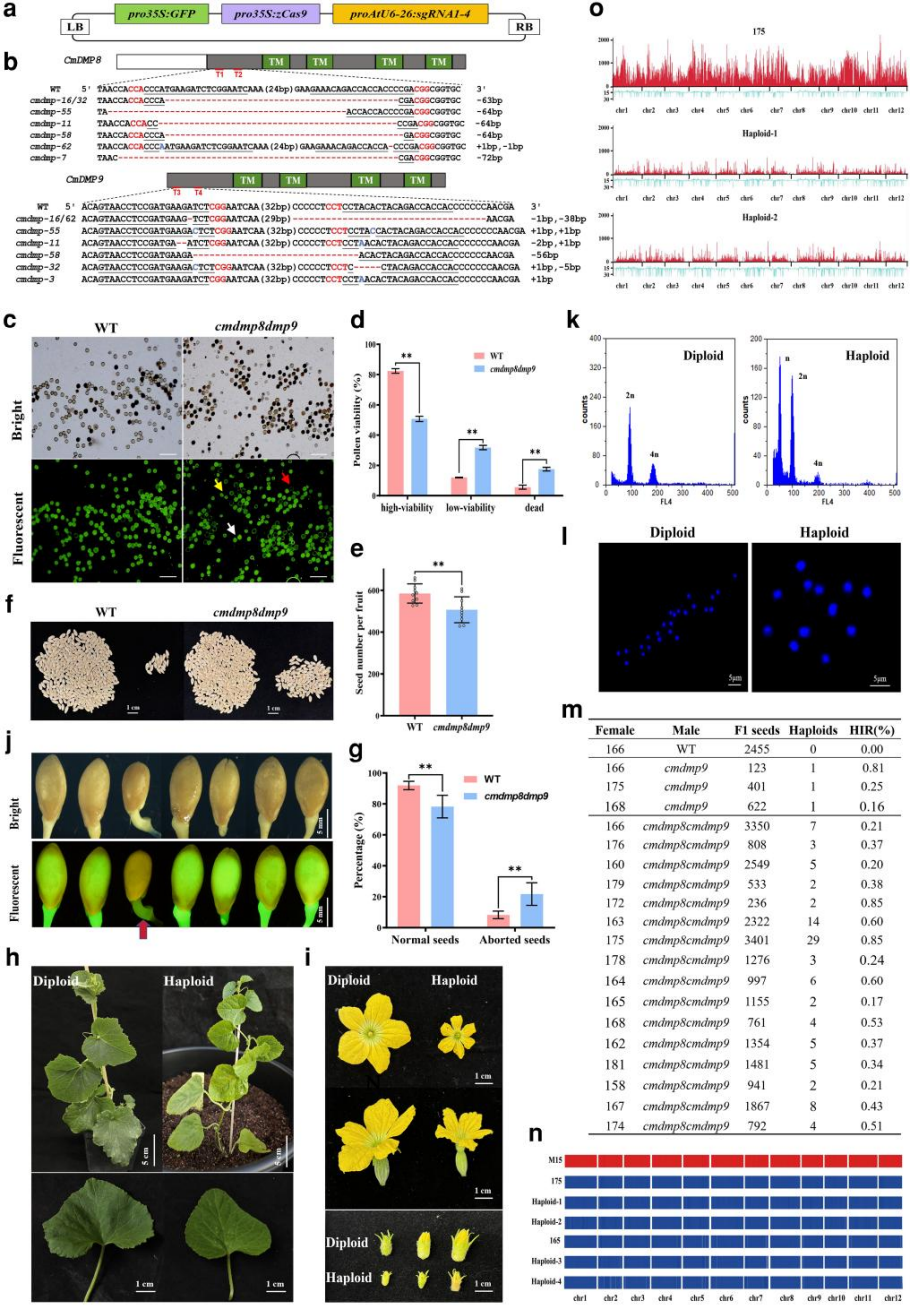
Maternal haploid induction in melon via editing of *CmDMP* genes. (a) Design of the CRISPR-Cas9 system for targeting *CmDMP* genes. (b) Schematic of the *CmDMP8* and *CmDMP9* gene structures and the genome editing strategy. Shaded blocks indicate the coding regions. Marked segments represent transmembrane domains (TM). Labelled lines denote the 4 sgRNA‑targeted regions. The sequences of wild type (WT) and mutant alleles in the T_1_ generation are presented below. (c and d) Analysis of pollen viability in WT and *cmdmp8cmdmp9* double mutants (T_1_). (c) Representative images from FDA (fluorescein diacetate) staining; scale bar, 200 μm. White, yellow, and red arrows indicate high viability, low viability, and dead pollen, respectively. (d) Quantification of pollen viability. Data are mean ± SD (n ≥ 5), **P < 0.01 (*t*-test). (e) Quantification of seed number per fruit. (f and g) Seed phenotypes per fruit in self-pollinated WT and T_1_ double mutants. Seed number comparison between WT and T_1_: Total seeds (WT = 5,848, T_1_ = 5,068); Normal seeds (WT = 5,380, T_1_ = 3,965); Aborted seeds (WT = 468, T_1_ = 1,103). Data are mean ± SD (n = 10), **P < 0.01 (*t*-test). (h and i) Phenotypic differences between diploid and haploid plants. (h) Representative plants and leaves; scale bar, 5 cm for whole plant; scale bar, 1 cm for leaf. (i) Representative floral organs (female flower, ovary, and male buds); scale bar, 1 cm. (j) GFP fluorescence-based haploid seed identification. The marked‑out arrow indicates the haploid; scale bar, 5 mm. (k and l) Ploidy verification of putative diploid and haploid plants. (k) Flow cytometry analysis. (l) Chromosome counting; scale bar, 5 μm. (m) HIR of different crosses in *CmDMP* mutants at the T_2_ generation. (n) SNP analysis confirms the maternal origin of haploids. The haploid lines 1 to 2 and 3 to 4 were derived from maternal parents ‘175' and ‘165', respectively. (o) Heterozygous SNP distribution in haploid lines 1 to 2 and their maternal parent ‘175' . The x-axis represents the genomic physical position, while the upper y‑axis indicates the heterozygous SNP count and the lower y‑axis indicates sequencing depth.

To determine the capability of *cmdmp8cmdmp9* mutants to induce haploid embryos in different genetic backgrounds, we crossed T_2_ homozygous mutants (homozygous for both mutant alleles and the GFP marker) as the male parent with 16 diverse melon lines. Because *DMP* inactivation may induce maternal haploids through single fertilization (Jacquier et al. 2020), we screened for maternal haploids among the cross progeny using the absence of green fluorescence as a primary indicator (Fig. 1j). Flow cytometry combined with chromosome counting was performed on 40 putative haploids and 10,171 putative diploids, confirming that the GFP marker enables 100% accurate identification of maternal haploids in melon (Fig. 1k and l; Table S5). Using this GFP marker, we then selected haploid seeds from crosses between 16 diverse melon genotypes and *cmdmp8cmdmp9* GFP-tagged mutant lines. Among 23,823 hybrid plants, 101 haploids were identified, yielding an average HIR of 0.42%, with individual rates ranging from 0.17% to 0.85% across different genetic backgrounds (Fig. 1m and Table S6). Additionally, 3 haploids were identified from *cmdmp9* single mutant-induced progeny, with an average HIR of 0.26% (Fig. 1m). To verify the maternal origin of these haploids, 4 representative seedlings were selected for whole-genome resequencing. Single-nucleotide polymorphism (SNP) analysis showed no paternally inherited SNPs in any of the seedlings (Fig. 1n), confirming that the *cmdmp8cmdmp9* double mutation induces pure maternal haploids. Furthermore, compared with their maternal parents, the obtained haploids exhibited high genome-wide homozygosity, with nearly all genomic regions homozygous (Fig. 1o and Figure S5).

In summary, we report the first maternal haploid induction in melon, filling a critical gap in cucurbits, as *DMP*-based haploid induction has been established in cucumber and watermelon (Yin et al. 2024; Tian et al. 2023). The GFP fluorescent marker enables reliable early screening of potential haploids at the radicle stage. The *cmdmp8cmdmp9* double mutant exhibited a notably higher HIR than the *cmdmp9* single mutant (Tables S4 and S6). While editing *CmDMP9* alone may suffice for routine breeding with moderate HIR demands, the double mutant is recommended for maximal efficiency across diverse genetic backgrounds. Recovered haploids survived well under standard greenhouse conditions, and chromosome doubling can be readily accomplished in the subsequent generation to produce DH lines. Given the laborious emasculation and cross-pollination in melon, the in vivo maternal haploid induction system constitutes a breakthrough in melon breeding.

## Supplementary Material

kiag552_Supplementary_Data

## Data Availability

All data in this study are available in this article and supplementary information.
