# Supplementary material for: In vivo efficient maternal haploid production in melon via knockout of CmDMP genes
Source: Plant Physiol. 2026 Aug 5;201(4):kiag552. doi: 10.1093/plphys/kiag552 (PMC13464498; doi:10.1093/plphys/kiag552)
Supplement: Supplementary file 1 [file kiag552_supplementary_data.zip › SupplementaryFigures.docx]

***In vivo* efficient maternal haploid production in melon via knockout of *CmDMP* genes**

**Supplementary Figures**

**Supplementary Figure S1**. Phylogenetic tree of DMPs in melon and other plant species.

**Supplementary Figure S2.** Subcellular localization of CmDMP:GFP fusion protein in epidermal cells of *Nicotiana benthamiana*; scale bar, 30 μm.

**Supplementary Figure S3.** The expression of *CmDMP8* and *CmDMP9* in different tissues.

**Supplementary Figure S4.** Phenotypes of WT and T_1_ generation *cmdmp8cmdmp9* double mutants.

**Supplementary Figure S5.** Heterozygous SNP distribution in haploid lines 3 to 4 and their maternal parent '165', with 'M15' (a highly homozygous inbred line) as the paternal inducer. Chromosomal distribution maps of heterozygous SNPs were generated for all 12 melon chromosomes. The x-axis represents the genomic physical position, while the upper y‑axis indicates the heterozygous SNP count and the lower y‑axis indicates sequencing depth.
