# Supplementary material for: In vivo efficient maternal haploid production in melon via knockout of CmDMP genes
Source: Plant Physiol. 2026 Aug 5;201(4):kiag552. doi: 10.1093/plphys/kiag552 (PMC13464498; doi:10.1093/plphys/kiag552)
Supplement: Supplementary file 1 [file kiag552_supplementary_data.zip › Supplemental Material.docx]

***In vivo* efficient maternal haploid production in melon via knockout of *CmDMP* genes**

**Supplementary materials and methods**

**Plant materials and growth conditions**

The *cmdmp* mutants used in this study were in the genetic background of the 'M15' melon cultivar, a highly homozygous inbred line, and 16 different melon varieties were employed as female parents in crossing to assess the HIR of these mutants. The T_0_ mutants were grown in the greenhouse under a photoperiod of 16 h light/8 h dark, with temperatures set at 28 °C (light) and 24 ℃ (dark). All crossed materials for HIR testing were grown under field conditions. Correspondingly, *Nicotiana benthamiana* seedlings were grown in the growth chamber (22 ℃, 16 h light/8 h dark photoperiod) at Nanjing Agricultural University, Nanjing, China.

**Identification of *DMP-like* in melon**

The amino acid sequences of ZmDMP and AtDMP8/9 were used as queries to blast the Melon Genome Database (http://cucurbitgenomics.org/organism/18), resulting in the identification of eight *DMP*-like candidate genes in melon (Table S2). Homologous *DMP* sequences of maize (*Zea mays*), *Arabidopsis* (*Arabidopsis thaliana*), alfalfa (*Medicago truncatula*), tomato (*Solanum lycopersicum*), tobacco (*Nicotiana tabacum*), oilseed rape (*Brassica napus*), cabbage (*Brassica oleracea*), cotton (*Gossypium hirsutum*), soybean (*Glycine max*), watermelon (*Citrullus lanatus),* and cucumber (*Cucumis sativus*) were downloaded from NCBI (https://www.ncbi.nlm.nih.gov/) using the GeneIDs reported in published papers (Zhong et al. 2019, 2020, 2022a, 2022b, 2024; Wang et al. 2022; Zhang et al. 2022a, 2022b; Li et al. 2022; Zhao et al. 2022; Long et al. 2024; Tian et al. 2023; Chen et al. 2023). A phylogenetic tree was constructed using MEGA11.0 with the neighbor-joining method (1000 bootstrap replications).

**Subcellular localization and fluorescent observation**

The subcellular localization analysis of CmDMP8 and CmDMP9 was conducted in *Nicotiana benthamiana* epidermal cells. The full-length coding sequences of *CmDMP8/9* (without stop codons) were cloned into the pGreen0029 vector, with primers listed in Table S7. The 35S::CmDMP8/9::GFP plasmid and the empty pGreen0029 vector were subsequently introduced into tobacco leaves via *Agrobacterium*-mediated transformation. The fluorescence of the 35S::CmDMP8/9::GFP fusion protein was examined using a confocal laser scanning microscope (Nikon A1 HD25, Japan). GFP was excited at 488 nm.

**Gene expression analysis**

Total RNA was extracted from melon 'M15' tissues, including root, stem, leaf, flower, anthers (0.3 mm and 0.5 mm), mature pollen, 4-DPA ovary, and 7-DPA ovary. cDNA was synthesized using the FastKing gDNA Dispelling RT SuperMix (TianGen Biotech, Beijing, China). RT-qPCR analysis was conducted in triplicate (three technical and three biological replicates) using SYBR Green chemistry (FastReal PreMix, TianGen) on a Bio-Rad CFX96 system. The *CmActin* gene served as the internal control, with gene-specific primers listed in Table S7. Relative expression of *CmDMP8/9* was calculated using the 2^−∆∆Ct^ method.

**CRISPR/Cas-mediated knockout of *CmDMPs***

The full-length DNA sequences of *CmDMP8* and *CmDMP9* were analyzed using the online tool CRISPR-P 2.0 (http://crispr.hzau.edu.cn/CRISPR2/). Based on the results from CRISPR-Direct, two target sites within the 5` region of each gene were selected for sgRNA design. The two designed sgRNAs were assembled into the binary vector pKSE402 at its *BsaI* restriction site, using the vector pCBC-DT1T2 as an intermediate. Stable transformation of the CRISPR/Cas9 expression cassette into 'M15' was performed using *Agrobacterium tumefaciens* strain GV3101, following a published method (Wan et al. 2023) with minor modifications. Putative transgenic plants were primarily selected based on green fluorescence and then genotyped using the high-throughput mutation detection (Hi-TOM) technique (Liu et al. 2019) to characterize mutations. The primer sequences used for PCR amplification are listed in Table S7.

**Pollen viability evaluation**

Mature pollen were collected from dehiscent anthers at 0 day post anthesis (DPA) of 'M15' and *cmdmp8cmdmp9* double mutants (T_1_ generation). Pollen viability was assayed using fluorescein diacetate (FDA) following the method of Zhong et al. (2019). The FDA working solution was prepared by mixing 100 µl FDA stock solution (0.5% (w/v) FDA in acetone) with 4.9 ml of 0.5 M sucrose solution. Viable pollen grains (exhibiting green fluorescence) were counted under an optical microscope (DP80, Olympus Corporation, Japan), and the viability percentage was calculated.

**Flow cytometry analysis**

Fresh young leaves (~2 cm^2^) were chopped with a fresh razor blade in 1 mL LB buffer (15 mM Tris, 2 mM Na_2_EDTA, 0.5 mM spermine tetrahydrochloride, 0.1% (v/v) Triton X-100, 15 mM β-mercaptoethanol, pH 7.5) on ice. The mixture was filtered through a 40-μm nylon mesh to isolate nuclei. The collected nuclei were resuspended in 450 μL of fresh LB buffer, supplemented with 25 μL of 1 mg/mL propidium iodide (PI) and 10 μL of 1 mg/mL RNase A. These samples were incubated on ice in the dark for 20 min and then analyzed by flow cytometry (CytoFLEX, Beckman Coulter, Inc., USA). Data were analyzed using CytExpert software (v1.0).

**Chromosome observation**

Young lateral roots (1–2 cm) were collected from both haploid and diploid plants. Root tips were excised, pretreated with an 8-hydroxyquinoline solution for 1 h 40 min, followed by rinsing with distilled water. Subsequently, they were then fixed in Carnoy’s fixative (glacial acetic acid: methanol, 1:3 [v/v]) for 24 h. Fixed root tips were then digested in an enzyme mixture containing 1% pectolyase Y23, 2% pectinase, 2% cellulase RS, and 4% cellulase R-10 at 37 °C for 50 min. The resulting protoplast suspension was dropped onto glass slides, gently squashed under a coverslip, and stained with DAPI. Chromosome images were acquired using a fluorescence microscope (Zeiss, Germany).

**SNP calling and breakpoint inference analysis**

To confirm the maternal origin of the putative haploids, genomic DNA from four haploid lines and their parental lines ('M15', '175', and '165') was subjected to whole-genome resequencing. Sequencing was performed on an Illumina NovaSeq X Plus platform (Shanghai Majorbio Bio-pharm Technology Co., Ltd.), generating an average depth of approximately 15×. Raw reads were filtered using the Fastp software (Chen et al. 2016). Clean reads were aligned to the melon reference genome (v3.6.1, available at the Cucurbit Genomics Database) using BWA-MEME (Jung et al. 2022). Single-nucleotide polymorphisms (SNPs) were carried out using GATK (Genome Analysis Toolkit, v4.x). The initial SNP set was filtered with the following criteria: (1) read depth between 5 and 30; (2) QUAL ≥ 40; (3) mapping quality (MQ) ≥ 30; and (4) located in non-repetitive genomic regions. The heterozygous SNP index was between 0.3 and 0.7, and the homozygous SNP index was ≤ 0.1 or ≥ 0.9. To determine the genetic origin of haploid lines, their SNP profiles were compared to the parental haplotypes. Crossover events were identified by scanning the genome with 0.1 Mb non-overlapping sliding windows and evaluating the similarity of SNP patterns between haploids and each parental haplotype.

**Supplementary** **References**

Chen X, Li Y, Ai G, Chen J, Guo D, Zhu Z, Zhu X, Tian S, Wang J, Liu M, et al. Creation of a watermelon haploid inducer line via *ClDMP3*-mediated single fertilization of the central cell. *Hortic Res*. 2023, 10: uhad081. https://doi.org/10.1093/hr/uhad081.

Chen X, Schulz-Trieglaff O, Shaw R, Barnes B, Schlesinger F, Källberg M, Cox AJ, Kruglyak S, Saunders CT. Manta: rapid detection of structural variants and indels for germline and cancer sequencing applications. *Bioinformatics*. 2016, 32: 1220–1222. https://doi.org/10.1093/bioinformatics/btv710.

Jung Y, Han D. BWA-MEME: BWA-MEM emulated with a machine learning approach. *Bioinformatics*. 2022, 38: 2404-2413. https://doi.org/10.1093/bioinformatics/btac137.

Liu Q, Wang C, Jiao X, Zhang H, Song L, Li Y, Gao C, Wang K. Hi-TOM: a platform for high-throughput tracking of mutations induced by CRISPR/Cas systems. *Sci China Life Sci*. 2019, 62: 1-7. https://doi.org/10.1007/s11427-018-9402-9.

Wan L, Wang Z, Zhang X, Zeng H, Ren J, Zhang N, Sun Y, Mi T. Optimised *Agrobacterium*-mediated transformation and application of developmental regulators improve regeneration efficiency in melons. *Genes*. 2023, 14: 1432. https://doi.org/10.3390/genes14071432.

Zhang J, Yin J, Luo J, Tang D, Zhu X, Wang J, Liu Z, Wang P, Zhong Y, Liu C, et al. Construction of homozygous diploid potato through maternal haploid induction. *aBIOTECH*. 2022a, 3: 163-168. https://doi.org/10.1007/s42994-022-00080-7.

Zhang X, Zhang L, Zhang J, Jia M, Cao L, Yu J, Zhao D. Haploid induction in allotetraploid tobacco using *DMPs* mutation. *Planta*. 2022b, 255(5): 98. https://doi.org/10.1007/s00425-022-03877-4.

Zhao X, Yuan K, Liu Y, Zhang N, Yang L, Zhang Y, Wang Y, Ji J, Fang Z, Han F, et al. *In vivo* maternal haploid induction based on genome editing of *DMP* in *Brassica oleracea.* *Plant Biotechnol J*. 2022, 20(12): 2242-2244. https://doi.org/10.1111/pbi.13934.

Zhong Y, Wang Y, Chen B, Liu J, Wang D, Li M, Qi X, Liu C, Boutilier K, Chen S. Establishment of a *dmp* based maternal haploid induction system for polyploid *Brassica napus* and *Nicotiana tabacum*. *J Integr Plant Biol*. 2022b, 64: 1281-1294. https://doi.org/10.1111/jipb.13244 .

Zhong Y, Yang M, Cheng D, Liu J, Han Q, Liu C, Qi X, Yan T, Teng L, Xv C, et al. Mutation of *GmDMP* genes triggers haploid induction in soybean. *bioRxiv*. 2024. https://doi.org/10.1101/2024.03.24.585499.
